# A chemical bonding based descriptor for predicting the role of anharmonicity induced by quantum nuclear effects in hydride superconductors

**DOI:** 10.1038/s41524-026-01973-7

**Published:** 2026-01-24

**Authors:** Francesco Belli, Eva Zurek, Ion Errea

**Affiliations:** 1 Department of Chemistry, State University of New York at Buffalo, Buffalo, NY USA; 2 Fisika Aplikatua Saila, Gipuzkoako Ingeniaritza Eskola, University of the Basque Country (UPV/EHU), Donostia/San Sebastián, Spain; 3 Centro de Física de Materiales (CFM-MPC), CSIC-UPV/EHU, Donostia/San Sebastián, Spain; 4 Donostia International Physics Center (DIPC), Donostia/San Sebastián, Spain

**Keywords:** Superconducting properties and materials, Physical chemistry

## Abstract

Quantum nuclear effects (QNEs) can significantly alter a material’s crystal structure and phonon spectra, impacting properties such as thermal conductivity and superconductivity. However, predicting a priori whether these effects will enhance or suppress superconductivity, or destabilize a structure, remains a grand challenge. Herein, we address this unresolved problem by introducing two possible descriptors, based upon the integrated crystal orbital bonding index (iCOBI) or the bond valence function, to predict the influence of QNEs on a crystal lattice’s dynamic stability, phonon spectra and superconducting properties. We find that structures with atoms in symmetric chemical bonding environments exhibit greater resilience to structural perturbations induced by QNEs, while those with atoms in asymmetric bonding environments are more susceptible to structural alterations, resulting in enhanced superconducting critical temperatures.

## Introduction

Achieving room-temperature and room-pressure superconductivity would benefit society in unforeseeable ways. Hydrogen-rich systems are the most promising compounds that could achieve this feat, as their strong electron phonon coupling (EPC) and large Debye temperatures allow them, in principle, to possess high superconducting critical temperatures, *T*_c_s, as originally proposed by Ashcroft^1,2^. Additionally, since the pairing mechanism in hydrides is presumed to be conventional, their superconducting properties can often be reasonably well predicted by theory, opening the door towards rational materials design. Indeed, many hydrides with high *T*_c_s have been synthesized, though at pressures impractical for applications. This includes H_3_S (*T*_c_ = 203 K near 150 GPa)^3^, LaH_10_ (*T*_c_ = 250–260 K near 200 GPa^4,5^), YH_4_ (*T*_c_ = ~ 88 K at 155 GPa^6^), YH_6_ (*T*_c_ = 220 K at 160–180 GPa^7,8^), YH_9_ (*T*_c_ = 240 K at 200 GPa^8^), CaH_6_ (*T*_c_ = 210-215 K at 160-172 GPa^9,10^), (La,Y)H_6_ and (La,Y)H_10_ (*T*_c_ = 237 and 253 K, respectively, both between 170-196 GPa^11^), as well as LaBeH_8_ (*T*_c_ = 110 K at 80 GPa^12^), (La,Ce)H_9−10_ (*T*_c_ = 176 K at 100 GPa^13^) and Y_0.5_Ce_0.5_H_9_ (*T*_c_ = 97–141 K between 98 and 200 GPa^14^).

As alluded to above, first-principles calculations based on density functional theory (DFT) have proven to be a valuable tool to direct experimental pursuits^15–17^, and many of the experimental discoveries have been anticipated by DFT calculations^18–22^. Furthermore, through DFT-based crystal structure prediction (CSP) methods, the landscape of hydrogen-host binary combinations has been meticulously explored. As a result, a clear understanding of the features enhancing superconductivity in electron-phonon mediated hydrogen-based superconductors has emerged^15,23,24^. Compounds with weakened covalent bonds, elevated hydrogen content, symmetric bonding configurations, and a high density of states (DOS) at the Fermi level (*E*_F_), with a large contribution arising from the hydrogen atoms, are likely to exhibit a high *T*_c_. Such insights are now being applied to direct the ab initio exploration of the phase diagrams of ternary and quaternary hydrides^25–31^. The hope is to further expand the list of predicted compounds (including those that are metastable), and scout for low-pressure-synthesizable systems, which would be necessary for practical applications of hydrogen-based superconductors.

The typical workflow^32^ for the ab initio prediction of hydrogen-based superconductors begins with identifying promising structures that correspond to (low energy) local or global minima in the Born-Oppenheimer energy surface (BOES). Next, the dynamic stability of the predicted compounds is determined by calculating the vibrational phonon frequencies in the harmonic approximation, and ensuring that no imaginary eigenvalues are present. This corresponds to a classic treatment of the nuclei, which are assumed to vibrate around their local minima. If, instead, the nuclei are treated as quantum particles, their positions are not clamped, but fluctuate around an average atomic position, known as a centroid. In this case, the phonon frequencies should be calculated from the second derivative of the total free energy with respect to the centroid positions, which includes the kinetic energy associated to the ionic quantum fluctuations. In hydrogen rich compounds, the harmonic classical treatment of the nuclei can yield predictions that differ vastly from those obtained with a proper quantum anharmonic treatment of the ions due to the light mass of hydrogen and the dynamical instabilities predicted at the harmonic level because of their potentially very large EPC^33^. The properties that are affected by quantum nuclear effects (QNEs) and anharmonicity in hydrides include: the optimized geometries, phonon frequencies, and perhaps most significantly for the current study, the superconducting behavior. These effects were shown to be extremely important for the first two high pressure hydrides that were synthesized, LaH_10_^33^ and H_3_S^34^. For example, DFT-based CSP searches found an $Fm\bar{3}m$ symmetry LaH_10_ structure to be the most stable at high pressures, but below 230 GPa other distorted variants were preferred, suggesting a complex BOES with many local minima^21,22,33,35^. However, within the harmonic approximation none of the proposed LaH_10_ phases (including $Fm\bar{3}m$) are dynamically stable below 230 GPa, so their *T*_c_ cannot be calculated. At the same time, experiments measured a *T*_c_ of 250 K from 137 to 218 GPa^3^ or 260 K at 188 GPa^4^ for a presumed LaH_10_ stoichiometry compound, resulting in a discrepancy between theory and experiment. This discrepancy could only be solved when QNEs were taken into account^33^. Since then, it has been shown that QNEs have a profound impact on the stability and superconducting properties of numerous high^36–38^ and ambient-pressure^39,40^ hydrides.

Though a number of computational techniques have been developed for treating QNEs and anharmonicity, the method that is often applied to superconducting hydrides is the stochastic self-consistent harmonic approximation (SSCHA)^41–45^. Recently, workflows that accelerate SSCHA calculations by leveraging machine-learned interatomic potentials have been developed^40,46,47^. Therefore, we can expect the number of SSCHA-based studies on hydrides to grow. However, the general trends of how QNEs and anharmonicity impact the superconducting properties of hydrogen-rich compounds is still not clear. In some cases they largely suppress superconductivity – a prime example being PdH where the strong anharmonicity of the material leads to an overestimation of the *T*_c_ by almost a factor of four (~ 35 K harmonic vs. ~ 10 K experiment)^39,41^, while in other situations, *T*_c_ can be considerably increased, such as in LaBH_8_, where QNEs enhance the *T*_c_ from about 100 K to 160 K at 100 GPa^48^.

Herein, by collecting a series of systems where QNEs and anharmonicity were treated through the SSCHA, we show the emergence of two possible scenarios. In the first, when inclusion of quantum nuclear effects does not perturb the geometry of a structure, these effects lower the *T*_c_. Conversely, when quantum treatment of the lattice changes its geometrical parameters, a concomitant increase in *T*_c_ is observed. Furthermore, we develop two chemical bonding based descriptors that can predict which of these scenarios describe a particular hydride. These descriptors, based upon a vector sum of either the integrated crystal orbital bonding index (iCOBI)^49^, or the bond valence function, are facile to compute, thereby enabling the automated classification of any arbitrary hydride into one of the two possible types. Our study results in a comprehensive, chemical bonding inspired understanding of the impact of QNEs and anharmonicity on the structures and phonon modes of hydrogen-based compounds, offering the possibility of discerning in advance their impact on superconducting properties.

## Results

### Two classes of structures

We begin by introducing the compounds that will be considered in our foray into the impact of QNEs on the properties of superconducting hydrides. Their structures and computed superconducting parameters have been taken from the cited literature, with the exception of the calculations outlined in the *Methods* section. As we will soon see, each one has its own story of how it is impacted by QNEs. As might be surmised by the reader, the way in which QNEs manifest themselves for the “Symmetric Bonding” (SB) systems described below differs markedly from how it influences compounds that belong to the “Asymmetric Bonding” (AB) class.

**Fig. 1:**
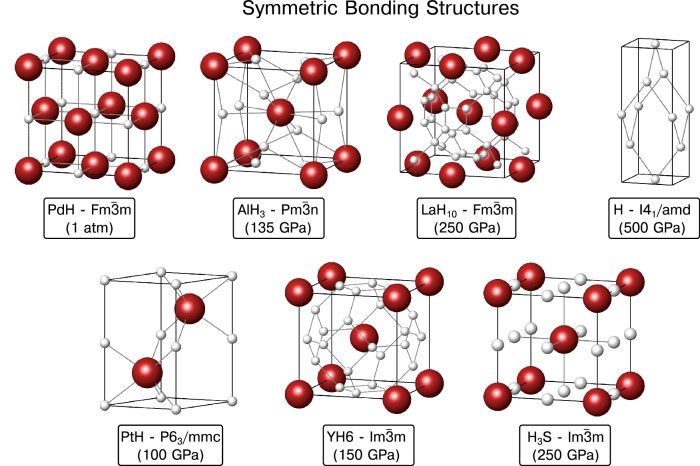
Symmetric bonding structures. Crystal structures of the SB class of compounds. Locally, these phases exhibit a high degree of symmetry in their bonding environments. Hydrogen atoms are white, and all other atom types are colored reddish-brown.

Figure 1 illustrates the SB crystal lattices and provides the pressure at which they have been computationally investigated. It turns out that the high symmetry of these lattices, and absence of free parameters in their Wyckoff positions, is key in determining the way in which they are impacted by QNEs. The first system to be described is PdH, which assumes a face-centered-cubic ($Fm\bar{3}m$) structure at ambient pressure (1atm = 1.01325 × 10^4^ Pa, in our calculations). A hallmark of conventional, or electron-phonon mediated, superconductors is that their *T*_c_ decreases upon substitution of a lighter isotope by a heavier one, because lighter atoms experience higher frequency vibrations. In a conventional superconductor with one type of atom of mass *M*, it is expected that *T*_c_ ∝ *M*^−*α*^, where *α* = 0.5 is the isotope coefficient. However, in PdH this coefficient is negative – a consequence of QNEs^39^ – such that its measured *T*_c_ is ~ 20% lower than that of its heavier brethren, PdD^50–56^. The next phase that we considered, AlH_3_-$Pm\bar{3}n$, is one of the first high-pressure hydrides to be theoretically^57^ and experimentally^58^ studied. Though calculations assuming classic nuclei predicted superconductivity, none could be measured down to 4 K^58^, and this discrepancy was shown to be a consequence of QNEs^36,59^. As already alluded to above, without the inclusion of QNEs LaH_10_-$Fm\bar{3}m$ is predicted to be dynamically unstable^60^ in a pressure range where it was synthesized and its superb superconducting properties were measured^4,5^, becoming dynamically stable with classical nuclei only above ~ 230 GPa. A hypothetical high-pressure phase of atomic hydrogen with *I*4_1_/*amd* symmetry computed to be the ground state above 577 GPa in the presence of QNEs with a *T*_c_ approaching 800 K at 2 TPa^61–64^ was also considered. As was a likely candidate of a synthesized hydride of platinum^65^ (PtH-*P*6_3_/*mmc*), which was calculated to have a *T*_c_ of 12 K at 90 GPa^65–67^, but whose superconductivity was shown to be suppressed upon the inclusion of QNEs^41^. In synthesized YH_6_-$Im\bar{3}m$ the measured *T*_c_ (224 K at 166 GPa^7^) is lower than predictions that do not treat the nuclei quantum mechanically (264 K at 120 GPa^68^). Turning to the experimentally synthesized H_3_S phase^3^, we consider in this class the $Im\bar{3}m$ structure, which is predicted to become enthalpically preferred over the *R*3*m* symmetry phase above 175 GPa^18,34^ when treating ions classically, but QNEs extend its range of dynamic stability down to ~ 100 GPa^34^ (though it may not be the thermodynamic minimum at this pressure). All the structures in the SB class are characterized by a high symmetry in their bonding environment about each atom.

**Fig. 2:**
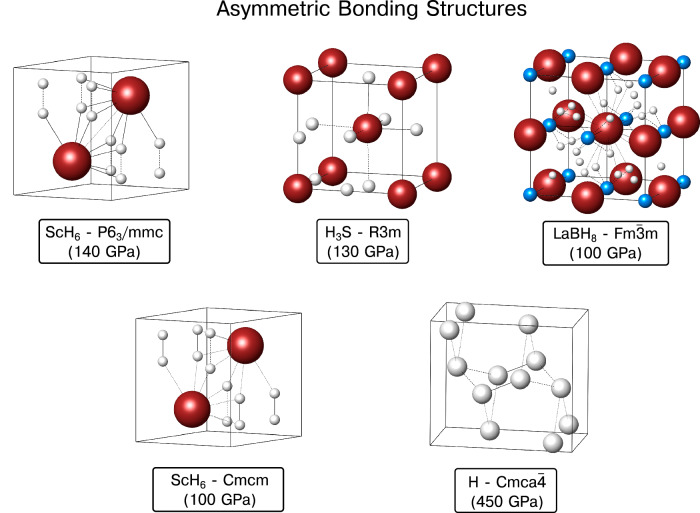
Asymmetric bonding structures. Crystal structures of the AB class of compounds. Locally, these phases exhibit a lower degree of symmetry in their bonding environments than the SB compounds (Fig. 1). Hydrogen atoms are white, B atoms in LaBH_8_ are blue, and all other atom types are colored reddish-brown. The H-*Cmca*-4 notation corresponds to the primitive cell, while here the conventional cell is shown.

The AB family (Fig. 2) includes two predicted ScH_6_ phases with H_2_ molecular units, which were thermodynamically preferred over the $Im\bar{3}m$ ScH_6_ clathrate structure below 275 GPa^69^. Diatomic hydrogen motifs comprise the hydrogenic sublattice in both, with the main difference between them being that the bond distances within the H_2_ molecules in the higher pressure phase (*P*6_3_/*mmc*) are the same, while in the lower pressure phase (*Cmcm*) they differ. Moreover, a distorted H_3_S structure, of *R*3*m* symmetry, which DFT calculations predict to be enthalpically preferred over $Im\bar{3}m$ H_3_S below 175 GPa was considered^19,70^. In the rhombohedral distortion three H-S distances become shorter than the others, thereby breaking the octahedral symmetry about the sulfur atoms that is present in the cubic phase. This distortion is associated with a large measured drop in *T*_c_, which was experimentally observed at 150 GPa signifying a small discrepancy between the predicted and measured pressure of the phase transition^3,71,72^. We also examined LaBH_8_, the first ternary hydride proposed that can be derived from LaH_10_ by removing the hydrogen atoms from the 8*c* Wyckoff position, and adding boron atoms to the 4*a* position^29,31,73^. A number of compounds with this structure type, but varying the identity of the electropositive and *p*-block elements, have been predicted^73,74^, and superconductivity has been measured in isotypic LaBeH_8_^12^. Finally, we considered the *Cmca*-4 phase of hydrogen^75^, composed of layered pairs of molecular hydrogen units in an ABAB hexagonal close packed stacking, which was predicted via static lattice calculations to be the most stable structure between 350 and 450 GPa^76^, or at 220 GPa and 300 K^77^, with a *T*_c_ of 242 K at 450 GPa^78^. More recent investigations, accounting for QNEs, have proposed a refined phase diagram where a *Cmca*-12 phase is the most stable between 410 and 577 GPa, while also suggesting that *Cmca*-4 is not the ground state at any pressure^62^. The local bonding in all these AB structures is less symmetric than those in the SB class.

### Quantum nuclear effects on the structural and superconducting properties

**Fig. 3:**
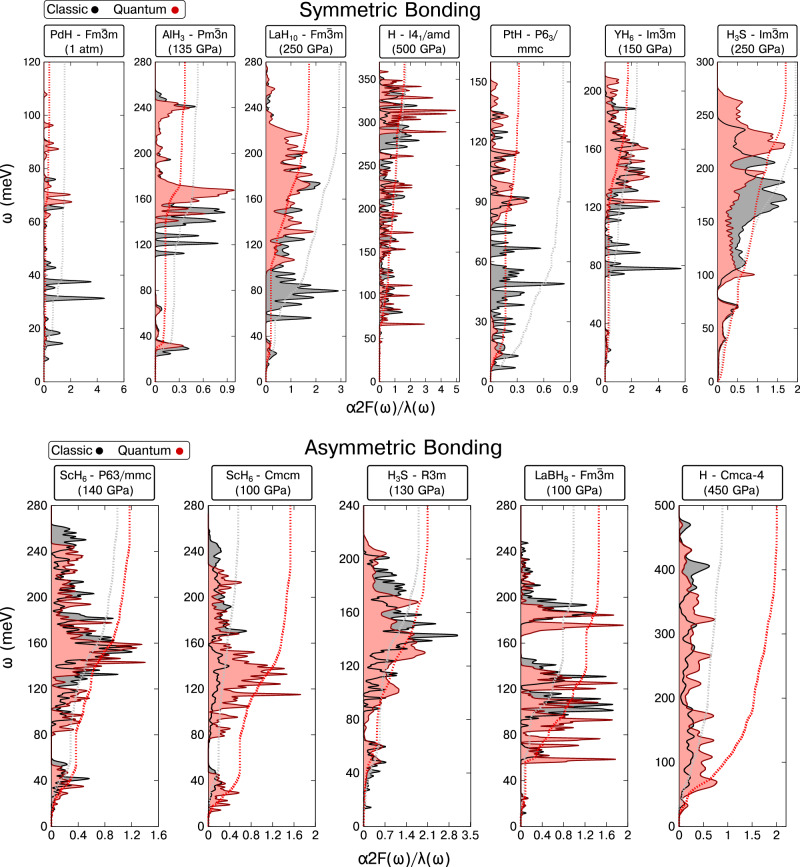
Eliashberg spectral functions. The Eliashberg spectral function (shaded curve, *α*^2^*F*(*ω*)), along with the integrated electron-phonon-coupling parameter (dashed line, *λ*(*ω*)), for the SB (upper panel) and AB (lower panel) structures illustrated in Figs. 1 and 2, respectively. Results for classical (quantum) nuclei are given in gray (red).

Now that we have described the structural characteristics of the considered compounds, let us examine the properties that are related to their superconducting response. The key quantity for calculating the *T*_c_ in conventional superconductors is the Eliashberg spectral function, *α*^2^*F*(*ω*), which can be understood as a phonon density of states, *F*(*ω*), weighted by the energy-dependent electron-phonon coupling, *α*(*ω*). Though *α*^2^*F*(*ω*) can, in principle, be measured (e.g. via inelastic tunneling spectroscopy^79^), it is often easier to calculate it via one of the various first-principles techniques that are available. Figure 3 plots the variation of *α*^2^*F*(*ω*) as a function of frequency computed with classical nuclei and neglecting anharmonicity (gray), as compared to a SSCHA calculation incorporating quantum anharmonic effects on the structure and the phonons at zero temperature (red). It is not uncommon for QNEs to expand the volume by a value that corresponds to ~ 10 GPa^33^, but for clarity here the two calculations are performed at the same volume. This comparison allows us to estimate, at the same time, the impact of QNEs and anharmonicity on the phonon spectra and the electron-phonon matrix elements. If we were to compare the *α*^2^*F*(*ω*) functions computed at the same pressure, it would not be possible to conclude if the difference between them was a result of the different volumes, or because of QNEs.

Due to their high symmetry and absence of free parameters in the Wyckoff positions, relaxation incorporating QNEs does not affect the structural coordinates of most of the SB compounds illustrated in Fig. 1 (PdH-$Fm\bar{3}m$, AlH_3_-$Pm\bar{3}n$, H-*I*4_1_/*amd*, PtH-*P*6_3_/*mmc*, YH_6_-$Im\bar{3}m$, and H_3_S-$Im\bar{3}m$). Because LaH_10_-$Fm\bar{3}m$ has a single free parameter, which is related to the 32f Wyckoff site occupied by a hydrogen atom, QNEs can, in principle, alter this atomic position. However, after SSCHA relaxation we observed that the variation in this coordinate is negligible, consistent with the other phases belonging to this class of hydrides.

Comparison of the red and gray *α*^2^*F*(*ω*) curves in the top panel of Fig. 3 reveals that QNEs tend to induce a blue shift of the phonon spectra in SB compounds, and this effect is most pronounced for the lower optical branches. Because these modes contribute significantly to the EPC constant, and *λ* ∝ *ω*^−2^, this phonon hardening tends to suppress *λ* (Supplementary Fig. 2), and also *T*_c_. At the pressures considered *T*_c_ decreases from 47 K to 5 K for PdH, 13.7 K to 3 K for AlH_3_, 260 K to 220 K for LaH_10_, 14.5 K to 0.4 K for PtH, 272 K to 247 K for YH_6_, and from 226 K to 190 K for H_3_S (Supplementary Table 2). The only exception to this trend is H-*I*4_1_/*amd*, where QNEs produce a red shift of the acoustic phonon branches, but a blue shift of the optical phonon branches, resulting in a nearly negligible effect on both *λ* and *T*_c_ (320 K versus 300 K), although when the volume expansion induced by QNEs is considered, the impact is a bit larger^80^.

Let us now consider the AB structures shown in Fig. 2, whose Eliashberg spectral functions are illustrated in the bottom panel of Fig. 3. Both of the ScH_6_ phases considered contain dihydrogen molecules that interact weakly with one another, and more strongly with scandium via charge transfer from the electropositive element to the hydrogen, coupled with H_2_*σ* → Sc d donation and Sc d → H_2_σ^*^ back-donation^81–83^. These metal-H_2_ interactions result in an elongation of the dihydrogen bond relative to what it would be in the molecular phase at the given pressure^83^. In ScH_6_-*P*6_3_/*mmc* at 140 GPa QNEs lengthen this bond even further, from 1.02 to 1.08 Å, with a concomitant red shift of the phonon modes and an increase in *T*_c_ from 88 K to 99 K. When the nuclei are treated classically, the ground state of ScH_6_ assumes *Cmcm* symmetry at 100 GPa. This phase can be derived from the higher-pressure *P*6_3_/*mmc* structure by breaking the symmetry and decreasing the distance between two sets of hydrogen atoms to 0.95 Å, and increasing the distance between another pair of H atoms to 1.28 Å. Therefore, the classical ScH_6_-*Cmcm* lattice can be better described as [Sc^2+^][2H^−^] ⋅ 2H_2_. However, quantum lattice fluctuations restore the broken symmetry, so all of the H-Sc bonds optimize to 1.08 Å at 100 GPa within the SSCHA, resulting in a fivefold increase in the *T*_c_ from 20 to 108 K.

A similar phenomenon occurs in H_3_S-*R*3*m*, which experiments suggest is preferred over the higher symmetry $Im\bar{3}m$ structure below 150 GPa^71^. At a volume corresponding to a classical pressure of 130 GPa, QNEs increase the pressure to ~ 140 GPa. Moreover, they symmetrize the H-S bonds, which range from 1.46 to 1.66 Å in the classic lattice, such that they all optimize to 1.56 Å in the quantum lattice. The change in these bond lengths shifts the Eliashberg spectral function to lower frequencies, with a concomitant increase in the *T*_c_ from 175 K to 214 K. Though, in principle, a similar result would be expected for LaH_10_ (at a pressure low enough so that the $Fm\bar{3}m$ phase is no longer the classical ground state^60^), we were unable to obtain a stable phonon spectra for any of the reported lower enthalpy structures, as all of them showed imaginary modes outside the zone center at the harmonic level.

Because LaBH_8_-$Fm\bar{3}m$ can be derived from LaH_10_, its structure also possesses a free parameter for the hydrogen atoms situated at the 32f Wyckoff positions. Within LaBH_8_ this parameter determines the B-H distance. Whereas in LaH_10_-$Fm\bar{3}m$ the inclusion of QNEs does not have a notable impact on the positions of these hydrogen atoms, the behavior is different within LaBH_8_-$Fm\bar{3}m$. Specifically, QNEs weaken and elongate the B-H bond from 1.366 to 1.385 Å, with a concomitant softening of the frequency associated with its vibration. As a result, the phonon spectra undergo a red shift and the *T*_c_ increases from 97 to 143 K at 100 GPa. Additionally, QNEs tend to destabilize this structure due to the stretching of the B-H bond, rendering it unstable at 77 GPa, a much higher pressure than the 35 GPa expected for classic nuclei^30,48^. This behavior is opposite to what has been found for H_3_S^60^ and LaH_10_^34^, where the pressure domain of dynamic stability is increased by QNEs. Finally, in the last AB structure considered, a molecular H_2_ phase with *Cmca* symmetry and four hydrogen atoms in the primitive cell, QNEs elongate the H-H distances from 0.78 to 0.83 Å at 400 GPa, weakening this bond with a concomitant red shift of the phonon modes, and a remarkable increase in the *T*_c_ from 109 to 258 K.

### A descriptor for the impact of quantum nuclear effects on *T*_c_

**Fig. 4:**
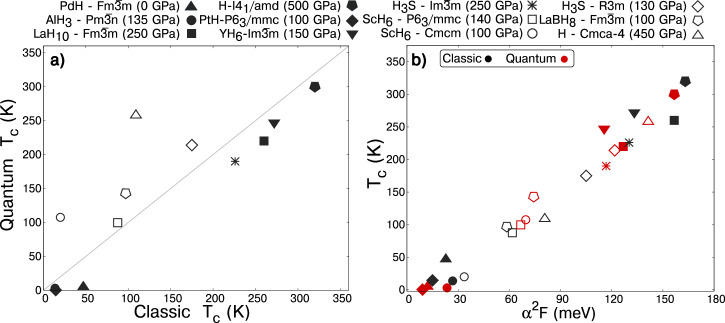
Classic and quantum critical temperatures. **a** The superconducting critical temperature, *T*_c_, computed for quantum nuclei versus classic nuclei for the SB (Fig. 1) and AB (Fig. 2) compounds studied herein. The open symbols (AB) fall above the diagonal line and represent those compounds whose quantum *T*_c_s are larger than their classic *T*_c_s, while the quantum *T*_c_s of the compounds denoted by the dark symbols (SB) are lower than their classical *T*_c_s. **b** The classical (black) and quantum (red) *T*_c_s of the studied compounds as a function of the Eliashberg spectral function, *α*^2^*F*(*ω*) (*a*^2^*F* = ∫ *α*^2^*F*(*ω*)d*ω*). The legend above the plots provides the symbol associated with each compound.

As described in the previous section, QNEs impact the geometries and structural features of the hydrides considered herein. Both these geometrical perturbations and anharmonicity can modify the frequencies associated with the phonon modes, which in turn affect the *T*_c_. Because symmetry lowering (e.g. Jahn-Teller or Peierls) distortions typically lead to the opening of gaps or pseudogaps, whereas symmetry raising transformations tend to increase the number of states that can participate in the EPC mechanism, QNE-induced structural changes could also vary the DOS at *E*_F_, with a concomitant effect on the electron-phonon matrix elements and therefore on the resulting *T*_c_. For the systems studied here belonging to the SB class, the practical absence of structural changes makes the DOS at *E*_F_ insensitive to QNEs, therefore any variations in *T*_c_ were mostly a result of the changes in the phonon frequencies. The systems in the AB class, however, undergo structural changes that in general tend to increase the DOS at the Fermi level, making the electron-phonon coupling larger. This is evident for H-*Cmca*-4 and ScH_6_-*Cmcm*, where the increased number of states at the Fermi level due to QNEs makes the value of the Eliashberg function much larger than for classical nuclei. Accordingly, in these cases the impact of QNEs is not only a shift of the phonon frequencies, but a more complex scenario in which the Fermi surface changes increase the coupling between the electrons and the ionic lattice. As highlighted by Fig. 4(a), QNEs typically decrease the *T*_c_ in SB compounds, whereas for the AB systems they tend to increase it, at times by a staggering factor of two (H-*Cmca*-4) or even four (ScH_6_-*Cmcm*) when their impact on the Fermi surface is remarkable.

Interestingly, we have found that the integral of the Eliashberg spectral function, defined as ${\alpha }^{2}F={\int }_{0}^{\infty }{\alpha }^{2}F(\omega )d\omega$, correlates very well with the *T*_c_ obtained for both classic harmonic and quantum anharmonic calculations (Fig. 4b). This finding indicates that *α*^2^*F* itself is a robust descriptor for analysing how QNEs impact *T*_c_. In fact, it correlates much better than the electron-phonon coupling constant, *λ*, and the average of the logarithmic phonon frequency, ${\omega }_{\log }$, the prefactor in the Allen-Dynes modified McMillan semiempirical equation used to calculate *T*_c_ (Supplementary Fig. 2).

**Fig. 5:**
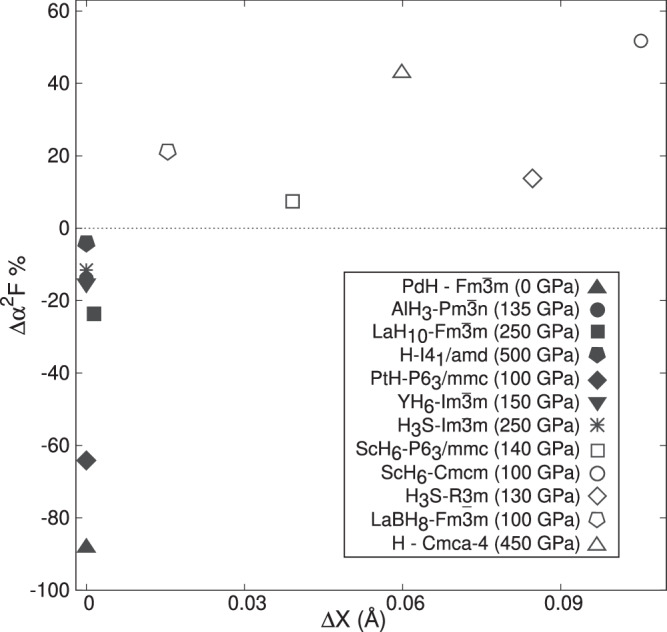
Quantum anharmonic displacement. The amount by which the integral of the Eliashberg spectral function (${\alpha }^{2}F={\int }_{0}^{\infty }{\alpha }^{2}F(\omega )d\omega$) differs, given as a percentage, between calculations that treat the nuclei as quantum particles versus classical particles, as a function of the mean atomic displacement, *ΔX*, defined in Equation (1). As shown in the legend, filled (open) symbols correspond to SB (AB) structures.

To better quantify the way in which QNEs affect each structure, we calculate the mean atomic displacements they impose as

1 $$\Delta X=\mathop{\sum }\limits_{a}^{{N}_{A}}\frac{| {\boldsymbol{\mathscr{R}}}_{{\rm{q}}}^{a}-{{\bf{R}}}_{{\rm{c}}}^{a}| }{{N}_{A}},$$

where ${\boldsymbol{\mathscr{R}}}_{{\rm{q}}}^{a}$ is the quantum anharmonic position calculated for atom *a*, ${{\bf{R}}}_{{\rm{c}}}^{a}$ is the position of the same atom as obtained from the minimum of the BOES, and *N*_*A*_ represents the number of atoms in the unit cell. Figure 5 plots the percentage change in *α*^2^*F* in going from the static to the quantum lattice as a function of *ΔX*, while the percentage change in *T*_c_ with respect to *ΔX* is plotted in Supplementary Fig. 3 panel c. It is clear that *α*^2^*F* can change dramatically, even when *ΔX* does not. For the SB structures, which are represented by full symbols, though the structure remains practically unaltered after the introduction of QNEs, a reduction for *α*^2^*F* is observed, implying a decreased *T*_c_. For the AB systems, represented by the empty symbols, the exact opposite is observed: the introduction of QNEs modifies the atomic parameters such that *ΔX* > 0 and *T*_c_ is increased. This analysis reveals that a comparison of the optimized geometries obtained assuming classic and quantum nuclei is sufficient to predict the way in which QNEs will affect the superconducting properties of hydrides. Our findings align with the analysis performed by Lucrezi et al. on LaBH_8_^47^, who concluded that QNEs give rise to two general phenomena that affect superconducting properties: one driven by structural changes and another related to phonon-phonon interactions resulting from anharmonicity. SB systems include those where the renormalization of the phonon-phonon interactions dominates over the structural perturbations, while the opposite phenomenon is characteristic of AB compounds.

However, to obtain *α*^2^*F* or *ΔX* a full geometry optimization and calculation of the phonon band structure and the EPC strength for both the classic and quantum lattice, the latter being particularly expensive even when accelerated using machine learning interatomic potentials, is still required. What we desire is a descriptor that predicts the way in which QNEs affect the geometric and superconducting properties of a structure that can be obtained knowing *only* the geometry of the classic lattice. One might be tempted to conclude that crystals with higher symmetries are likely to be perturbed less by QNEs than ones with lower symmetries. However, such a rule of thumb would be incorrect, as the structurally similar LaH_10_ (SB) and LaBH_8_ (AB) compounds both assume the $Fm\bar{3}m$ spacegroup. Another example where spacegroups would not help differentiate between the two types of systems is PtH and ScH_6_ at 140 GPa, which both adopt *P*6_3_/*mmc* symmetry, the former a member of the SB family and the latter a member of the AB family.

Since the total number of symmetry operations of the spacegroup and the total number of free parameters in its Wyckoff sites is insufficient to predict a priori how QNEs will impact a structure, another metric must be found. Because our observations suggested that the local bonding environments about the atoms in a phase are key for predicting how it will be affected by QNEs, we desire to employ an observable able to quantify the strength of the interatomic interactions. Towards this end, we first introduced a vector quantity, ***V***_*x*_, computed for each atom *x*. This vector is the sum of a generic scalar function *f*(*x*, *α*) defining a value for all of the interactions between an atom *x* and its neighboring atoms *α*, and the function is weighted by a unit vector, ***i***_*xα*_, which denotes the direction of each interaction. The result is summed over all neighbors for which the *f*(*x*, *α*) values fall above a user-defined threshold, and divided by the number of interactions considered, *B*_*x*_, as

2 $${{\boldsymbol{V}}}_{x}=\frac{1}{{B}_{x}}\mathop{\sum }\limits_{\alpha =1}^{{B}_{x}}f(x,\alpha ){{\boldsymbol{i}}}_{x\alpha }.$$

The idea is that, due to the vectorial summation of interaction strengths, ***V***_*x*_ can capture the asymmetry of the local bonding environment around the atom *x* and quantify the effect of QNEs on the structure. However, ***V***_*x*_ exhibits redundancy due to the symmetry of the system: atoms located on equivalent Wyckoff positions are expected to have identical magnitudes of ***V***_*x*_. To simplify the analysis and reduce the number of variables, we investigated *S*_*a*_ = ∑_*x*∈*a*_∣***V***_*x*_∣/*N*_*a*_ as the average value for all the atoms, *x*, belonging to the same Wyckoff position *a*. We defined this variable as the *symmetry index* or *symmetry parameter*. The symmetry index is expected to be very small or zero for an atom that is in a completely symmetric bonding environment, whereas larger values of *S*_*a*_ correlate with a greater degree of local bonding asymmetry around the atom.

A natural question arises: is it possible to find an appropriate function, *f*(*x*, *α*), that can be used to distinguish SB from AB structures based on the symmetry parameters calculated for each symmetry-inequivalent atom in the optimized classical lattice, ${S}_{a}^{{\rm{c}}}$? Moreover, would the symmetry parameters for quantum nuclei, ${S}_{a}^{{\rm{q}}}$, differ between these two classes of compounds? To answer this question, we tested multiple functions. We first considered the vector weighted sum of the interatomic distances about an atom, and the Crystal Orbital Hamiltonian Population integrated to *E*_F_ (iCOHP)^84^ between atom pairs, both summed over the unit cell, as descriptors (Supplementary Table 6 and 7). However, neither approach demonstrated sufficient predictive power. Analyzing interatomic distances alone is insufficient because while one distance might correspond to a value typical of a single bond for one atom pair, for another atom pair it may be typical of a non-bonding or even a repulsive interaction. Though the iCOHP is sensitive to the bond strength, our tests showed that it was not able to predict which class LaH_10_ and H-*Cmca*-4 would fall into (Supplementary Table 6).

Subsequently, we considered the local interatomic bonding strengths as calculated via the integral of the crystal orbital bonding index (iCOBI)^49^ for pairs of atoms. The iCOBI is a generalization of the bond index according to Wiberg and Mayer, which is directly related to the classic bond order, adapted to periodic systems. For a single bond, such as between two carbon atoms in diamond, an iCOBI of 1 would be expected, whereas in an ionic system, such as between the Na^+^ and Cl^−^ atoms in the rocksalt phase, iCOBI should be near 0. Previous computations yielded 0.95 and 0.09 for these cases^49^, in-line with our expectations. In a mixed covalent and ionic system, a value between 0 and 1 is therefore likely to be found.

In the high pressure hydrides the interaction between hydrogen atoms is expected to yield iCOBIs that fall between 1 (for an H_2_ molecule) and 0 (between hydridic hydrogen, H^−^, or atomic hydrogen, H, and a hydrogen atom in any other hydrogenic motif). When *p*-block elements form weak covalent bonds with the hydrogen atoms, such as in H_3_S, the iCOBI is expected to be notably larger than 0, but smaller than 1. For example, in $Im\bar{3}m$ H_3_S at 270 GPa the iCOBI between the S and H atoms was computed to be 0.34, indicating a bond order of roughly 1/3^85^. While the interaction between electropositive elements and hydrogen is expected to be primarily ionic, we note that covalent (dative) interactions such as those arising from donation of orbitals from bonding states to a vacant d-orbital on the metal atom and back-bonding from an occupied metal d-orbital to antibonding hydrogenic states (*e.g*. H_2_*σ* → metal d, and metal d → H_2_σ^*^)^81–83^ are likely to be non-negligible resulting in non-zero iCOBIs. Importantly, we hypothesized that as long as the individual iCOBIs are not equal to naught, a vector sum of them about an atom can provide information about the symmetry of the atom’s local bonding environment. Calculations were performed testing the symmetry index metric obtained using two different cutoff values for the iCOBIs. In the first we removed iCOBIs whose values were less than 0.05 in the summation to remove all hydrogen interactions beyond 1.4 Å, and in the second cutoff all iCOBI values below 0.018 were removed to retain as many interactions as possible, but remove all of the noise associated to the orbital projections (Supplementary Tables 8 and 9). For both cutoffs the iCOBIs were able to differentiate between the SB and SA classes of hydrides.

The iCOBI, however, is limited. Though it can determine the covalent component of the bond strength, it does not at all quantify the ionic bonding, which may also impact the local bonding symmetry. To address this limitation, we have as an alternative investigated the bond valence function^86^, defined such that its sum around each atom is equal to the atom’s oxidation state (or valence). This function has the form:

3 $$BV(r)={e}^{\frac{{r}_{0}-r}{b}},$$

where *r*_0_ and *b* represent parameters for the bond length of the interaction and decay factor, while *r* is the actual length of the interaction in question, respectively. A ready to use list of parameter for *r*_0_, and *b* has been proposed and is available in the literature^86^.

This list however is not complete and lacks information for some interactions including those for hydrogen-hydrogen bonds. Since we are concerned with hydrogen-rich systems at various pressures, we expect that, due to pressure variations and the electronic charge donation from metals in the structure, the ideal values for the *r*_0_ and *b* parameters for the hydrogen-hydrogen interactions will be highly system-dependent. We have therefore adjusted the parameters of the bond valence function for the hydrogen-hydrogen interactions by calibrating them against the iCOBI to to match the proper *r*_0_ and the decay factor *b*. Although the iCOBI captures just the covalent part of the bond order, we expect the ionic contribution for the H-H bond valence to be small, so that our approximation, while not perfect, remains reasonable for the H-H interactions. In the case of hydrogen, we identified three distinct decay behaviors: one associated with pure hydrogen phases, one corresponding to hydrogen in clathrate-like configurations (LaH_10_, YH_6_), and one related to negatively charged hydrogen atoms, such as in the ScH_6_ system, where we computed Bader charges of -0.11*e* to -0.2*e* on the hydrogen atoms, that were donated by scandium. For other cases in which hydrogen-hydrogen interactions were not detected through the iCOBI the parameters of *r*_0_ = 0.74 (corresponding to the standard distance of the H_2_ molecule) and *b* = 0.37 (most commonly used decay factor) were used. The parameters employed are listed in Supplementary Fig. 4. The bond valence function based symmetry indices were calculated for each symmetry inequivalent atoms in the hydrides illustrated in Figs. 1, 2, and the results are provided in Table 1. The analysis was performed by imposing a distance cutoff of 1.4 Å for the hydrogen-hydrogen interactions and a cutoff for all values of the bond valence function of 0.1. The cutoffs were chosen to include all the significant interactions, while at the same time removing those originating from the long range decay of the bond valence function.

**Table 1:** Symmetry index based on bond valence function

Structure	Atom	Wyckoff letter	${S}_{a}^{{\rm{c}}}$	${S}_{a}^{{\rm{q}}}$
Symmetric Bonding (SB)				
PdH - $Fm\bar{3}m$ (1 atm)	Pd	4a	0	0
	H	4b	0	0
AlH_3_ - $Pm\bar{3}m$ (135 GPa)	Al	2a	0	0
	H	6c	0	0
LaH_10_ - $Fm\bar{3}m$ (250 GPa)	La	4b	0	0
	H	8c	0	0
	H	32f	0.016	0.063
H - *I*4_1_/*amd* (500 GPa)	H	4b	0	0
PtH - *P*6_3_/*mmc* (100 GPa)	Pt	2d	0	0
	H	2a	0	0
YH_6_ - $Im\bar{3}m$ (150 GPa)	Y	2a	0	0
	H	12d	0	0
H_3_S - $Im\bar{3}m$ (250 GPa)	S	2a	0	0
	H	6b	0	0
Asymmetric Bonding (AB)				
ScH_6_ - *P*6_3_/*mmc* (140 GPa)	Sc	2d	0	0
	H	12k	0.145	0.131
ScH_6_ - *Cmcm* (100 GPa)	Sc	4c	0.002	0 (2d)
	H	16h	0.176	0.120 (12k)
	H	8f	0.093	0.120 (12k)
H_3_S - *R*3*m* (130 GPa)	S	3a	0.117	0
	H	9b	0.175	0
LaBH_8_ - $Fm\bar{3}m$ (100 GPa)	La	4b	0	0
	B	4a	0	0
	H	32f	0.085	0.079
H - *Cmca*-4 (450 GPa)	H	8f	0.114	0.079

The symmetry inequivalent atoms and their Wyckoff positions in the SB (Fig. 1) and AB (Fig. 2) structures along with the corresponding symmetry index computed treating the nuclei as classical, ${S}_{a}^{{\rm{c}}}$, and quantum, ${S}_{a}^{{\rm{q}}}$, objects calculated with a cutoff for the H-H distances of 1.4 Å and a secondary cutoff of 0.1 on the bond valence function (Equation 3) to remove the long range decay for all other interactions. The Wyckoff parameters given in parenthesis for ScH_6_—*Cmcm* correspond to the positions of these atoms in the higher symmetry ScH_6_ - *P*6_3_/*mmc* phase.

Examination of Table 1 clearly shows that for SB structures both the classic and quantum asymmetry parameters are the same and are either zero, or very nearly zero, for each symmetry inequivalent atom. In fact, the only non-zero value that was calculated was for the hydrogen atoms at the 32f Wyckoff position in LaH_10_, which has a free parameter. The value of ${S}_{a}^{{\rm{c}}}$ for this Wyckoff position, 0.016, is much smaller than the non-zero values computed for any of the AB structures. Thus, we suggest that the classical lattice symmetry index, ${S}_{a}^{{\rm{c}}}$, should be less than or equal to 0.02 for every atom within hydrides that belong to the SB family, that is those where QNEs do not alter the structure, but where the phonon modes are shifted to higher frequencies because of the phonon-phonon interactions. These combined effects reduce *λ*, as well as *α*^2^*F*, leading to a concomitant decrease in *T*_c_ in SB systems. Our analysis rationalizes why LaH_10_ belongs to the SB family: despite the intrinsic freedom of the 32f Wyckoff site, the local bonding environment about these atoms is sufficiently symmetric to ensure that the atomic positions are not strongly altered by QNEs.

For AB structures, on the other hand, at least one of the symmetry inequivalent atoms possesses a significant ${S}_{a}^{{\rm{c}}}$. Interestingly, the symmetry parameters for all but two of the heavy atoms are zero, in-line with the finding that QNEs do not perturb their positions during the relaxation, such that ${S}_{a}^{{\rm{q}}}$ is also zero. The two specific exceptions are the S and Sc atoms within H_3_S-*R*3*m* and ScH_6_ - *Cmcm*, where the classical symmetry index of 0.117 and 0.002 decreased to zero during structural relaxation with the SSCHA, resulting in a symmetric environment about these atoms. In-line with the expectation that the lighter hydrogen atoms should have a higher proclivity to be perturbed by QNEs than the heavy atoms, they had significantly larger ${S}_{a}^{{\rm{c}}}$ values (0.085–0.176), which all decreased substantially when their lattices were optimized with the SSCHA. Additionally, we note that LaBH_8_-$Fm\bar{3}m$ has the 32f site occupied by a hydrogen atom as in LaH_10_-$Fm\bar{3}m$. However, the two systems are part of a different class. This highlights that interatomic interactions play a more relevant role than crystal symmetry in determining how QNEs perturb a structure. All these results are summarized in (Supplementary Fig. 3 panel a and b) where we highlight the correlation between the mean value of ${S}_{a}^{{\rm{c}}}$ and the displacement *ΔX*, and the variation of *Δ* *T*_c_ with respect to the mean value of ${S}_{a}^{{\rm{c}}}$. Results obtained with the iCOBI displayed the same trends, though the non-zero values of the symmetry indices differed slightly, as shown in Supplementary Tables 8 and 9.

The method proposed here is intended to resolve subtle variations arising from quantum nuclear effects in hydrogen rich materials. Moreover, we can conclude that compounds with at least one large ${S}_{a}^{{\rm{c}}}$ value tend to increase the symmetry in their local bonding environment upon structural relaxation with QNEs, in particular around the hydrogen atoms, shifting the phonon modes to lower frequencies. These effects serve to increase *λ*, as well as *α*^2^*F*, and the concomitant *T*_c_, in stark contrast to what is observed in SB systems. More importantly the results shown in Supplementary Table 7 highlights that these effects cannot be captured by pure geometrical descriptors. Specifically, Supplementary Table 7 proposes a similar method to the Baur distortion index^87^ considering the average deviation of the distances. Similarly, the quadratic elongation and bond-angle variance index^88^, or the bond angle variance^89^ are inappropriate in this context, as they cannot capture the strength of the interactions. Furthermore, they consider the divergence of structural parameters from ideal reference geometries, which are not reliable at high pressure.

In our analysis, both the iCOBI and the bond-valence method^90^ are capable of classifying the systems according to how they are perturbed by QNEs. The two approaches are conceptually similar, each with its own limitations. The iCOBI offers a more straightforward implementation, as it is derived directly from orbital projections, but it is restricted to capturing only the covalent bonding component. In contrast, the bond-valence method accounts for covalent and ionic interactions, but for the high-pressure systems that assume structures and compositions not found at 1 atm, requires careful parameter tuning through additional procedures.

## Discussion

This work investigates how quantum nuclear effects (QNEs) and the anharmonicity they induce can alter the structural and superconducting properties of hydrogen-based superconductors. We introduce a symmetry bonding index or parameter, *S*_*a*_, for each symmetry inequivalent atom, which is based upon a vector weighted sum of either the bond valence function (Equation 3), or the integrated crystal orbital bonding indices (iCOBIs) between this atom and others. An *S*_*a*_ value of zero corresponds to an atom in a nearly perfect symmetric bonding configuration, whereas larger values of *S*_*a*_ are associated with non-negligible deviations from ideal symmetry. This index highlights that the local bonding environment plays a more important role than crystal symmetry in determining the behavior of a structure perturbed by QNEs, as highlighted by the different response of LaH_10_-$Fm\bar{3}m$ and LaBH_8_-$Fm\bar{3}m$. Furthermore, the index can be computed for lattices of classical nuclei optimized within the Born-Oppenheimer approximation, ${S}_{a}^{{\rm{c}}}$, as well as relaxations that treat the nuclei quantum mechanically, ${S}_{a}^{{\rm{q}}}$. Importantly, we find that the classical results are sufficient to predict the way in which QNEs will impact the properties of both ambient pressure and high pressure hydride superconductors. Thus, ${S}_{a}^{{\rm{c}}}$, which can be calculated knowing only the positions of the classical lattice, is a robust and quick-to-compute descriptor that can predict how QNEs and anharmonicity affect the geometry of a compound and its superconducting behavior.

Two families of structures were identified and analyzed. In symmetric bonding, or SB systems, ${S}_{a}^{{\rm{c}}}$ was calculated to be zero for most atom types, and we suggest that it should not exceed 0.02 for the bond valence (and 0.01 for the iCOBI) for any atom belonging to phases in this class. QNEs do not perturb the geometries of SB compounds, but anharmonicity lowers the pressure at which they are predicted to become dynamically unstable, with a consequent blue shift of their phonon spectra, which is stronger for the lower optical branches. This perturbation of the phonon modes results in a decrease of the *T*_c_. In asymmetric bonding, or AB systems, on the other hand, ${S}_{a}^{{\rm{c}}}$ is large for at least one of the symmetry inequivalent atoms, meaning that these phases have a lower degree of local bonding symmetry. Structural relaxation with QNEs attempts to restore the local symmetry, often (but not always) resulting in smaller ${S}_{a}^{{\rm{q}}}$ values. This geometric perturbation of the quantum lattice destabilizes the structure, softens the phonon modes, and enhances *T*_c_, sometimes by an impressive factor of 2–4 when QNEs increase the density of states at the Fermi level. We expect the introduced classical asymmetry parameter will become a powerful tool in the a priori prediction of the effect of QNEs on the geometries, phonon modes and critical temperatures of hydride superconductors, and its quantum counterpart will be used to understand the magnitude of these effects for specific lattices and atom types.

To conclude, we speculate that the symmetry index, based on the iCOBI or the bond valence function, could offer additional value in characterizing atomic interactions and crystal properties. In particular, it might offer insights on the behavior of systems under pressure variations or strain, or for the study of the local symmetry about atoms potentially in amorphous systems, fluids and glasses. These ideas lay foundations for further studies.

## Methods

### Dataset and computational details

The structural parameters and Eliashberg spectral functions, both with and without SSCHA-treated QNEs, were obtained from the literature. The dataset includes the following compounds: PdH at ambient pressure^39^, AlH_3_ at 135 GPa^36^, LaH_10_ at 250 GPa^60^, a phase of atomic hydrogen (*I*4_1_/*amd* at 500 GPa^91^), PtH at 100 GPa^41^, YH_6_ at 150 GPa^7^, H_3_S-$Im\bar{3}m$ at 250 GPa and 130 GPa^34^, ScH_6_-*P*6_3_/*mmc* at 140 GPa^37^, ScH_6_-*Cmcm* at 100 GPa, H_3_S-*R*3*m* at 130 GPa^34^, LaBH_8_ at 100 GPa^48^, and a phase of molecular hydrogen (*Cmca*-4 at 450 GPa^92^). Moreover, additional calculations were performed to obtain data for ScH_6_-*Cmcm* at 100 GPa, as described below. All these listed spacegroups and pressures correspond to the classical calculation, excluding the potential impact on the structure and the estimated pressure of QNEs.

In most of these investigations^7,34,36,37,41,48,60,91,92^, the geometries were initially optimized within the Born-Oppenheimer approximation at the specified pressures. The SSCHA was subsequently applied to relax the atomic positions including QNEs and anharmonicity, while keeping the unit cell shape and dimensions fixed. This approach allows for a direct comparison of the phonons and the Eliashberg spectral functions at identical volumes; however, the pressures determined via SSCHA are higher from those computed using classical nuclei due to the extra contribution arising from the QNEs. In contrast, the comparative analysis between the classical and quantum structures for ScH_6_-*Cmcm* at 100 GPa and ScH_6_-*P*6_3_/*mmc* at 140 GPa was conducted by relaxing the structures with the SSCHA also at 100 and 140 GPa, respectively, which implies that the lattices in the comparison are different in these two cases.

The ScH_6_-*Cmcm* phase was identified by displacing the atoms according to the eigenvectors of the imaginary *E*_2*g*_ symmetry mode at *Γ* computed for ScH_6_-*P*6_3_/*mmc* at 100 GPa^37^, followed by a subsequent structural relaxation. The DFT calculations for ScH_6_-*Cmcm* were performed with the plane-wave Quantum ESPRESSO package^93,94^. We employed the Perdew-Burke-Ernzerho^95^ parameterization of the exchange-correlation potential (PBE-GGA), along with ultrasoft pseudo-potentials that treated 11 electrons of scandium in the valence, with cutoffs for the wavefunctions and density chosen as 1088 eV and 10,884 eV, respectively. The Brillouin zone integration in the self-consistent calculations were performed with a first-order Methfessel-Paxton smearing with a broadening of 0.27 eV, and a **k**-mesh with a spacing of 2*π* × 0.011 Å^−1^. The harmonic phonon calculations were performed on a **q**-mesh with a spacing of 2*π* × 0.056 Å^−1^, making use of density functional perturbation theory^96^. The electron-phonon interaction was calculated on a **k**-mesh with a spacing of 2*π* × 0.0083 Å^−1^, with a Gaussian smearing of 0.11 eV to approximate the Dirac deltas, and with the same phonon grid that was used in the phonon calculations. The *T*_c_ for all compounds were recalculated using the isotropic Migdal-Eliashberg^97^ formalism with a Coulomb repulsion parameter, μ^*^, of 0.1.

In this formalism, the Eliashberg spectral function for the quantum anharmonic calculations reported in this manuscript was calculated as

4 $${\alpha }^{2}F(\omega )=\frac{1}{2\pi N({\epsilon }_{F}){N}_{q}}\mathop{\sum }\limits_{\mu {\bf{q}}}\frac{{\gamma }_{\mu }({\bf{q}})}{{\omega }_{\mu }({\bf{q}})}\delta [\omega -{\omega }_{\mu }({\bf{q}})],$$

where

5 $${\gamma }_{\mu }({\bf{q}})=\frac{\pi }{{N}_{k}}\mathop{\sum }\limits_{{\bf{k}}nm}\mathop{\sum }\limits_{ab}\frac{{\epsilon }_{\mu }^{a}({\bf{q}}){\epsilon }_{\mu }^{b}{({\bf{q}})}^{* }}{\sqrt{{M}_{a}{M}_{b}}}{d}_{{\bf{k}}n,{\bf{k}}+{\bf{q}}m}^{a}{d}_{{\bf{k}}n,{\bf{k}}+{\bf{q}}m}^{b* }\delta ({\varepsilon }_{{\bf{k}}n}-{\varepsilon }_{F})\delta ({\varepsilon }_{{\bf{k}}+{\bf{q}}n}-{\varepsilon }_{F})$$

is the phonon linewidth associated with the electron-phonon interaction for the phonon mode *μ* at the wave vector **q**. In these two equations *N*(*ϵ*_F_) is the density of states at the Fermi energy, *N*_*q*_ and *N*_*k*_ represent, respectively, the number of phonon **q** and electron **k** points, *M*_*a*_ and *M*_*b*_ are the atomic masses associated with the *a*^th^ and *b*^th^ atoms, *ω*_*μ*_(**q**) and ${\epsilon }_{\mu }^{a}({\bf{q}})$ represent phonon frequencies and eigenvectors, and ${d}_{{\bf{k}}n,{\bf{k}}+{\bf{q}}m}^{a}$ represents the electron-phonon matrix element of the form

6 $${d}_{{\bf{k}}n,{\bf{k}}+{\bf{q}}m}^{a}=\langle {\bf{k}}n\left|\delta {V}_{{\rm{KS}}}/\delta {R}^{a}({\bf{q}})\right|{\bf{k}}+{\bf{q}}m\rangle .$$

Equation (6) is the vertex of the scattering process from a Kohn-Sham state $\left\langle {\bf{k}}n\right|$ with momentum **k** and band index *n*, to the state $\left|{\bf{k}}+{\bf{q}}m\right\rangle$ with momentum **k** + **q** and band index *m*, mediated by the variation of the Kohn-Sham potential *V*_KS_ with respect to the displacement of the *a*^th^ atom at position *R* with modulation (**q**).

The electron-phonon properties in Equations (4) and (5) were computed for both the classical harmonic and quantum anharmonic cases. In the classical case, these properties were evaluated using atomic positions obtained from self-consistent relaxation. Conversely, in the quantum anharmonic case, the atomic positions were taken from the SSCHA relaxation. The superconducting properties in the quantum anharmonic framework were determined using phonon frequencies (*ω*_*ν*_(**q**)) and eigenvectors (${\epsilon }_{\mu }^{a}({\bf{q}})$) obtained via SSCHA, while the electron-phonon matrix elements (${d}_{{\bf{k}}n,{\bf{k}}+{\bf{q}}m}^{a}$) were derived from the classical dynamical matrices, using the atomic structure relaxed within SSCHA.

### Bonding analysis

To analyze the bonding, DFT calculations for all of the structures in our dataset were performed using the Vienna ab initio Simulation Package (VASP)^98^, with the PBE-GGA and an energy cut-off of 800 eV. The number of valence electrons treated explicitly and their electronic configurations are the following: H 1s^1^, B 2s^2^2p^1^, Al 3s^2^3p^1^, S 3s^2^3p^4^, Sc 3d^2^4s^1^, Y 4s^2^5s^2^4p^6^4d^2^, Pd 4d^9^5s^1^, Pt 5d^9^6s^1^, and La 5s^2^6s^2^5p^6^5d^1^. The core electrons were treated with the projector augmented wave (PAW) method^99^. The reciprocal space was sampled using a *Γ*-centered Monkhorst-Pack^100^**k**-mesh with a spacing of 2*π* × 0.016 Å^−1^ with an electronic smearing of 0.2 eV. The integrated Crystal Orbital Bonding Index (iCOBI)^49^, which is a quantification of the extent of covalent bond formation, was calculated for atom pairs using the LOBSTER^101^ code with the standard basis set proposed and reported in Supplementary Table 3. The results did not change when a larger basis set was employed. In order to asses the appropriateness of the exchange-correlation functional for the computation of the iCOBI, we have repeated the calculation for the molecular *Cmca*-4 hydrogen phase using the R^2^SCAN meta-GGA^102^. No difference was observed between the two results.

## Supplementary information

Supplementary Information

## Data Availability

All data used in this study is available on request from the authors.Quantum ESPRESSO and SSHCA are open-source codes available at https://www.quantum-espresso.org and https://sscha.eu, respectively. The program used to calculate the symmetry index is available at https://github.com/FlemkMeserath/QuantumObservable. VASP is a propietary software.

## Code availability

Quantum ESPRESSO and SSCHA are open-source codes available at https://www.quantum-espresso.organd https://sscha.eu, respectively. The program used to calculate the symmetry index is available at https://github.com/FlemkMeserath/QuantumObservable. VASP is a proprietary software.
