# Supplementary material for: A chemical bonding based descriptor for predicting the role of anharmonicity induced by quantum nuclear effects in hydride superconductors
Source: NPJ Comput Mater. 2026 Jan 24;12(1):100. doi: 10.1038/s41524-026-01973-7 (PMC12935538; doi:10.1038/s41524-026-01973-7)
Supplement: Supplementary file 1 — Supplementary Information [file 41524_2026_1973_MOESM1_ESM.pdf]

Supplementary Information: A chemical bonding based descriptor for predicting the role of anharmonicity induced by quantum nuclear effects in hydride superconductors

Francesco Belli,[†] Eva Zurek,[*,†] and Ion Errea[*,‡,¶,§]

†*Department of Chemistry, State University of New York at Buffalo, Buffalo, NY 14260-3000, USA*

‡*Fisika Aplikatua Saila, Gipuzkoako Ingeniaritza Eskola, University of the Basque Country (UPV/EHU), Europa Plaza 1, 20018 Donostia/San Sebastián, Spain*

¶*Centro de Física de Materiales (CFM-MPC), CSIC-UPV/EHU, Manuel de Lardizabal Pasealekua 5, 20018 Donostia/San Sebastián, Spain*

§*Donostia International Physics Center (DIPC), Manuel de Lardizabal Pasealekua 4, 20018 Donostia/San Sebastián, Spain*

E-mail: ezurek@buffalo.edu; ion.errea@ehu.eus

Contents

1 Phonons and Eliashberg Function of ScH_6-$Cmcm$ **3**

2 Structural Parameters **4**

3 Superconductivity **5**

4 Definition of the iCOHP and iCOBI **7**

5 Bonding Analysis **8**

References **16**

1 Phonons and Eliashberg Function of ScH_6-$Cmcm$

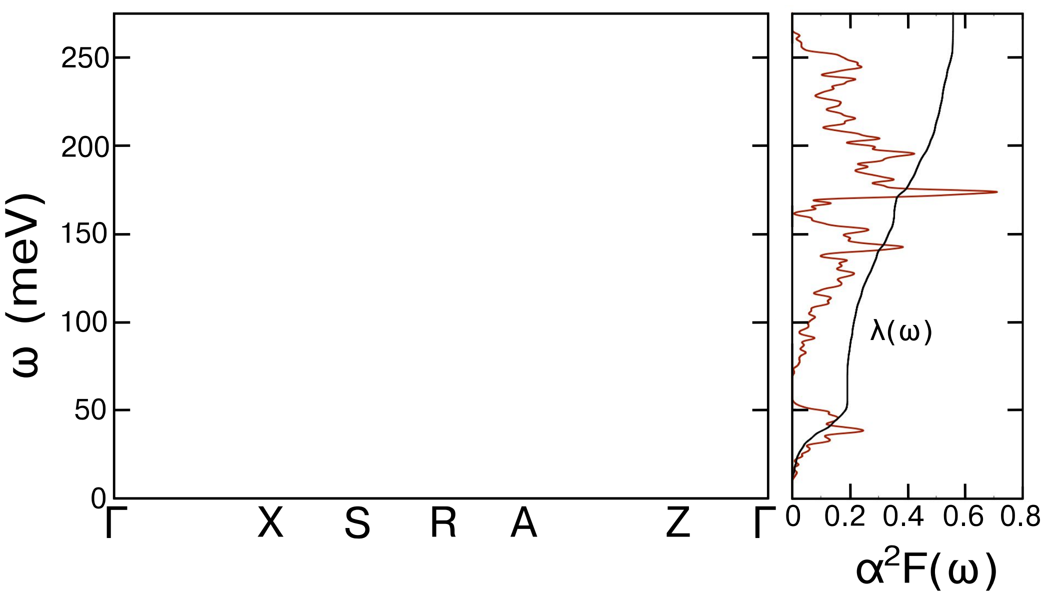

Supplementary Figure 1: **Phonon spectrum and Eliashberg spectral function.** The classic phonon spectra for the ScH_6 - $Cmcm$ phase at 100 GPa (left), and the variation of the Eliashberg spectral function, $\alpha^2F(\omega)$, along with the integral of the electron phonon coupling parameter, $\lambda(\omega)$, with the frequency (right).

2 Structural Parameters

Supplementary Table 1: **Structural parameters.** The cell parameters and atomic positions for the considered compounds obtained when the nuclei were treated as classic particles.

Structure	Parameters	Atom	Wyckoff Site	Coordinate
PdH - $Fm\bar{3}m$ (1 atm)	a = 4.132 Å	Pd	4a	0 0 0
		H	4b	0.50 0.50 0.50
AlH_3 - $Pm\bar{3}n$ (135 GPa)	a = 3.003 Å	Al	2a	0 0 0
		H	6c	0.50 0.25 0
LaH_{10} - $Fm\bar{3}m$ (250 GPa)	a = 3.424 Å	La	4b	0.50 0.50 0.50
		H	8c	0.25 0.25 0.25
		H	32f	0.10 0.10 0.10
H - $I4_1/amd$ (500 GPa)	a = 1.120 Å<br>c = 3.110 Å	H	4b	0.50 0.50 0
PtH - $P6_3/mmmc$ (100 GPa)	a = 2.710 Å	Pt	2d	0.33 0.67 0.75
	c = 4.576 Å	H	2a	0 0 0
	γ = 120°			
YH_6 - $Im\bar{3}m$ (150 GPa)	a = 4.103 Å	Y	2a	0 0 0
		H	12d	0.25 0 0.50
H_3S - $Im\bar{3}m$ (250 GPa)	a = 3.007 Å	S	2a	0 0 0
		H	6b	0.50 0.50 0
ScH_6 - $P6_3/mmc$ (140 GPa)	a = 3.406 Å	Sc	2d	0.67 0.33 0.25
	c = 4.255 Å	H	12k	0.67 0.83 0.13
	γ = 120°			
ScH_6 - $Cmcm$ (100 GPa)	a = 3.434Å	Sc	4c	0 0.33 0.75
	b = 6.215 Å	H	16h	0.75 0.43 0.36
	c = 4.433 Å	H	8f	0.83 0.66 0.89
H_3S - $R3m$ (130 GPa)	a = 3.116 Å	S	3a	0 0 0
		H	9b	0.53 0.01 0.01
$LaBH_8$ - $Fm\bar{3}m$ (200 GPa)	a = 5.577 Å	La	4b	0.50 0.50 0.50
		B	4a	0 0 0
		H	32f	0.15 0.15 0.15
H - $Cmca$-4 (450 GPa)	a = 1.534 Å<br>b = 2.674 Å<br>c = 2.360 Å	H	8f	0 0.37 0.43

3 Superconductivity

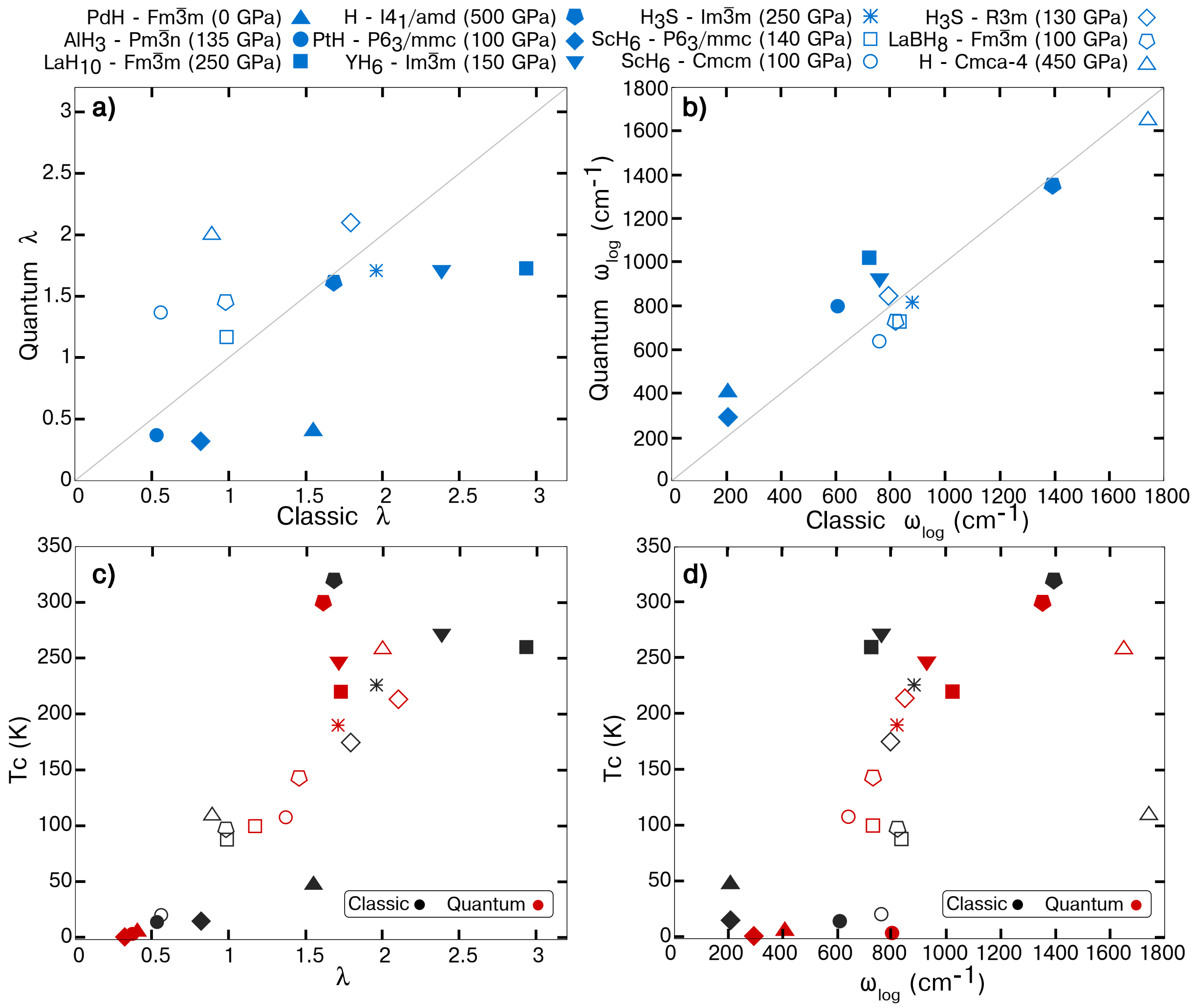

Supplementary Figure 2: **Correlation between critical temperatures and superconducting parameters.** A comparison of (a) the electron phonon coupling parameter, λ, and (b) the logarithmic average phonon frequency, $\omega_{\log}$, between the classical and quantum structures. The line drawn in (a) and (b) corresponds to $y = x$. (c) The classic (black) and quantum (red) values of T_c as a function of λ. (d) The classic (black) and quantum (red) T_c as a function of $\omega_{\log}$.

Supplementary Table 2: **Superconducting parameters.** The electron phonon coupling (λ), logarithmic average phonon frequency (ω_{log}), and superconducting critical temperature (T_{c}), for the classical and quantum structures divided into Symmetric Bonding (SB), and Asymmetric Bonding (AS) families, respectively on the top and bottom parts of the table.

Structure	Classic			Quantum		
	λ	ω_{log} (cm^{-1})	T_{c} (K)	λ	ω_{log} (cm^{-1})	T_{c} (K)
PdH - $Fm\bar{3}m$ (1 atm)	1.55	205	47	0.40	405	5
AlH_3 - $Pm\bar{3}n$ (135 GPa)	0.53	607	13.7	0.37	799	3.08
LaH_{10} - $Fm\bar{3}m$ (250 GPa)	2.93	722	260	1.72	1021	220
H - $I4_1/amd$ (500 GPa)	1.68	1393	320	1.61	1351	300
PtH - $P6_3/mmc$ (100 GPa)	0.82	205	14.5	0.32	291	0.378
YH_6 - $Im\bar{3}m$ (150 GPa)	2.38	760	272	1.71	926	247
H_3S - $Im\bar{3}m$ (250 GPa)	1.96	879	226	1.71	817	190
ScH_6 - $P6_3/mmc$ (140 GPa)	0.99	833	87.5	1.16	728	99.6
ScH_6 - $Cmcm$ (100 GPa)	0.56	759	20	1.36	639	108
H_3S - $R3m$ (130 GPa)	1.79	793	175	2.10	846	214
$LaBH_8$ - $Fm\bar{3}m$ (100 GPa)	0.98	819	97	1.46	729	143
H - $Cmca$-4 (450 GPa)	0.89	1742	109	2.00	1650	258

4 Definition of the iCOHP and iCOBI

The Crystal Orbital Hamiltonian Population[1] (COHP) and the Crystal Orbital Bonding Index[2] (COBI) are derived starting from the assumption that the LCAO wave functions ($\Phi_j(\mathbf{k})$) and the band functions ($\psi_j(\mathbf{k}, \mathbf{r})$) must closely resemble each other, despite their different definition. Such a $\mathbf{k}$-dependent LCAO function $\Phi_j(\mathbf{r})$ can be expressed for the jth band by combining atom-centered, orthonormal one-electron functions (orbitals) $\phi_\mu(\mathbf{r})$ with coefficients $c_{j\mu}^{\mathbf{k}}$ as:

$$\mid \Phi_j(\mathbf{k})\rangle = \sum_\mu c_{j\mu}^{\mathbf{k}} \mid \phi_\mu\rangle \approx \mid \psi_j(\mathbf{k})\rangle \tag{1}$$

Under this assumption the COHP is defined as the real part of the projection of the plane wave Hamiltonian over the atomic orbitals at a specific energy (E) as

$$\text{pCOHP}_{\mu\nu}(E, \mathbf{k}) = \sum_j f_{j\mathbf{k}} \mathbb{R}[P_{\mu\nu j}(\mathbf{k}) H_{\mu\nu}(\mathbf{k})] \delta(\varepsilon_j(\mathbf{k}) - E), \tag{2}$$

where $f_{j\mathbf{k}}$ represents the occupation of band j and wavevector $\mathbf{k}$, $H_{\mu\nu}(\mathbf{k})$ defines the projected elements of the Hamiltonian with eigenvalues $\varepsilon_j(\mathbf{k})$

$$H_{\mu\nu}(\mathbf{k}) = \sum_j \langle\phi_\mu \mid \psi_j(\mathbf{k})\rangle \varepsilon_j(\mathbf{k}) \langle\psi_j(\mathbf{k}) \mid \phi_\nu\rangle, \tag{3}$$

and $P_{\mu\nu j}(\mathbf{k})$ represents the elements of the projected density matrix for band j and every $\mathbf{k}$-point

$$P_{\mu\nu j}(\mathbf{k}) = \langle\phi_\mu \mid \psi_j(\mathbf{k})\rangle \langle\psi_j(\mathbf{k}) \mid \phi_\nu\rangle. \tag{4}$$

A similar form is obtainable for the COBI as

$$\text{pCOBI}_{\mu\nu}(E, \mathbf{k}) = \text{BO}_{\mu\nu} \sum_j \mathbb{R}[P_{\mu\nu j}(\mathbf{k})] \delta(\varepsilon_j(\mathbf{k}) - E) \tag{5}$$

where $\text{BO}_{\mu\nu}$ is the Coulson's mobile bond order.[3]

The iCOHP and the iCOBI are then obtained by summing all over the **k**-points and by integrating up to the Fermi energy as

$$\mathrm{iCOHP}_{\mu\nu} = \int^{\varepsilon_F} \sum_{\mathbf{k}} \mathrm{pCOHP}(E, \mathbf{k})_{\mu\nu} \mathrm{d}E \tag{6}$$

and

$$\mathrm{iCOBI}_{\mu\nu} = \int^{\varepsilon_F} \sum_{\mathbf{k}} \mathrm{pCOBI}(E, \mathbf{k})_{\mu\nu} \mathrm{d}E \tag{7}$$

5 Bonding Analysis

Supplementary Table 3: **Basis set.** List of the basis set used for each atom in the iCOBI analysis.

Element	Basis Functions
H	1s
B	2s 2p
Al	3s 3p
S	3s 3p
Sc	3d 4s
Y	4d 4p 4s 5s
Pd	4d 5s 5p
Pt	6s 6p 5d 4f
La	4f 5d 5p 5s 6s

Supplementary Table 4: **iCOBI interactions for symmetrically bonded (SB) systems.** Distances and integrated crystal orbital bond indices (iCOBIs) for the listed atom pairs in the SB compounds for which iCOBI $\geq$ 0.05. The results were the same for the classical and quantum lattice for all of the compounds, except for LaH_{10} where the two sets of values are separated by a forward slash.

Structure	Atom Pair	Distance (Å)	iCOBI
PdH - $Fm\bar{3}m$	Pd-Pd	2.922	0.135
	Pd-H	2.066	0.138
AlH_3 - $Pm\bar{3}n$	Al-H	1.678	0.161
LaH_{10} - $Fm\bar{3}m$	La - H	2.096 / 2.096	0.122 / 0.091
	La - H	2.053 / 2.015	0.115 / 0.145
	H - H	0.972 / 1.087	0.120 / 0.106
	H - H	1.254 / 1.165	0.055 / 0.072
H - $I4_1/amd$	H - H	0.985	0.144
PtH - $P6_3/mmc$	Pt - H	1.938	0.064
YH_6 - $Im\bar{3}m$	Y - H	2.017	0.124
	H - H	1.275	0.053
H_3S - $Im\bar{3}m$	H - S	1.534	0.347

Supplementary Table 5: **iCOBI interactions for asymmetrically bonded (AB) systems.** The distances and integrated crystal orbital bond indices (iCOBIs) for the listed atom pairs in the AB compounds calculated for both the classic and quantum lattices for which iCOBI $\geq$ 0.05.

Structure	Atom Pair	Classic		Quantum	
		Distance (Å)	iCOBI	Distance (Å)	iCOBI
ScH_6 - $P6_3/mmc$	H-H	1.020	0.240	1.068	0.220
	Sc-H	1.777	0.178	1.783	0.178
	Sc-H	1.896	0.137	1.884	0.141
	Sc-Sc	2.897	0.091	2.910	0.086
ScH_6 - $Cmcm$	H-H	0.950	0.330	1.083	0.233
	H-H	1.275	0.117	1.083	0.233
	Sc-H	1.826(5)	0.169	1.833	0.174
	Sc-H	1.826(8)	0.195	1.833	0.174
	Sc-H	1.847	0.203	1.942	0.138
	Sc-H	2.032	0.102	1.942	0.138
	Sc-Sc	2.935	0.092	2.986	0.084
H_3S - $R3m$	H-S	1.456	0.463	1.559	0.347
	H-S	1.661	0.231	1.559	0.347
$LaBH_8$ - $Fm\bar{3}m$	B-H	1.409	0.281	1.454	0.291
	B-La	2.788	0.164	2.788	0.150
	La-H	2.285	0.094	2.281	0.097
H - $Cmca$-4	H-H	0.782	0.424	0.832	0.358
	H-H	1.034	0.109	1.071	0.091
	H-H	1.158	0.051	1.079	0.086

Supplementary Table 6: **iCOHP based symmetry index.** The symmetry inequivalent atoms and their Wyckoff positions in the SB and AB structures along with the corresponding symmetry index computed with the iCOHP, $\mathbf{V}(\text{iCOHP})_x = \frac{1}{B_x}\sum_{\alpha=1}^{B_x}\text{iCOHP}(x,\alpha)\mathbf{i}_{x\alpha}$ and $S(\text{iCOHP})_x = |\mathbf{V}(\text{iCOHP})_x|$ (see main text), treating the nuclei as classical, $S(\text{iCOHP})_a^{\text{c}}$, and quantum, $S(\text{iCOHP})_a^{\text{q}}$, objects. The iCOHP based results are not able to distinguish between SB and AB structures as the value of $S(\text{iCOHP})_a^{\text{c}}$ for the hydrogens at the 32f site in LaH_{10} is greater than the values computed for AB H - $Cmca$-4 structure. The Wyckoff parameters given in parenthesis for ScH_6 - $Cmcm$ correspond to the symmetry site and multiplicity of the equivalent atoms in the higher symmetry ScH_6 - $P6_3/mmc$ phase. The values of the iCOHP used in the analysis have been selected using a cutoff of 0.5 (eV/bond), so as to consider the same amount of interactions reported in the iCOBI analysis.

Structure	Atom	Wyckoff letter	$S(\text{iCOHP})_a^{\text{c}}$ (eV/bond)	$S(\text{iCOHP})_a^{\text{q}}$ (eV/bond)
Symmetric Bonding				
PdH - $Fm\bar{3}m$	Pd	4a	0	0
	H	4b	0	0
AlH_3 - $Pm\bar{3}n$	Al	2a	0	0
	H	6c	0	0
LaH_{10} - $Fm\bar{3}m$	La	4b	0	0
	H	8c	0.01	0.01
	H	32f	0.22	0.08
H - $I4_1/amd$	H	4b	0	0
PtH - $P6_3/mmc$	Pt	2d	0	0
	H	2a	0	0
YH_6 - $Im\bar{3}m$	Y	2a	0	0
	H	12d	0	0
H_3S - $Im\bar{3}m$	S	2a	0	0
	H	6b	0	0
Asymmetric Bonding				
ScH_6 - $P6_3/mmc$	Sc	2d	0	0
	H	12k	0.52	0.48
ScH_6 - $Cmcm$	Sc	4c	0	0 (2d)
	H	16h	0.33	0.47 (12k)
	H	8f	0.77	0.47 (12k)
H_3S - $R3m$	S	3a	0.68	0
	H	9b	1.03	0
$LaBH_8$ - $Fm\bar{3}m$	La	4b	0	0
	B	4a	0	0
	H	32f	0.70	0.73
H - $Cmca$-4	H	8f	0.17	0.09

Supplementary Table 7: **Interatomic distance based symmetry index.** The symmetry inequivalent atoms and their Wyckoff positions in the SB and AB structures along with the corresponding symmetry index computed with the magnitudes of the interatomic distances, $\mathbf{V}(\mathrm{d})_x = \frac{1}{B_x}\sum_{\alpha=1}^{B_x} \mathrm{d}(x,\alpha)\mathbf{i}_{x\alpha}$ and $S(\mathrm{d})_x = |\mathbf{V}(\mathrm{d})_x|$(see main text), treating the nuclei as classical, $S(\mathrm{d})_a^{\mathrm{c}}$, and quantum, $S(\mathrm{d})_a^{\mathrm{q}}$, objects. Here $\mathrm{d}(x,\alpha)$ is the interatomic distance between atoms x and α. The distance based results are not able to distinguish between SB and AB structures as the value of $S(\mathrm{d})_a^{\mathrm{c}}$ for the hydrogens at the 32f site in LaH_{10} is greater than the $S(\mathrm{d})_a^{\mathrm{c}}$ for both atoms in the AB H_3S - $R3m$ structure. The Wyckoff parameters given in parenthesis for ScH_6 - $Cmcm$ correspond to the symmetry site and multiplicity of the equivalent atoms in the higher symmetry ScH_6 - $P6_3/mmc$ phase. The atom pairs whose interatomic distances were considered in this analysis correspond to those considered for the iCOBI analysis.

Structure	atom	Wyckoff letter	$S(\mathrm{d})_a^c$ (Å)	$S(\mathrm{d})_a^q$ (Å)
Symmetric Bonding				
PdH - $Fm\bar{3}m$	Pd	4a	0	0
	H	4b	0	0
AlH_3 - $Pm\bar{3}n$	Al	2a	0	0
	H	6c	0	0
LaH_{10} - $Fm\bar{3}m$	La	4b	0	0
	H	8c	0	0
	H	32f	0.18	0.03
H - $I4_1/amd$	H	4b	0	0
PtH - $P6_3/mmc$	Pt	2d	0	0
	H	2a	0	0
YH_6 - $Im\bar{3}m$	Y	2a	0	0
	H	12d	0	0
H_3S - $Im\bar{3}m$	S	2a	0	0
	H	6b	0	0
Asymmetric Bonding				
ScH_6 - $P6_3/mmc$	Sc	2d	0	0
	H	12k	0.29	0.32
ScH_6 - $Cmcm$	Sc	4c	0.38	0 (2d)
	H	16h	0.41	0.32 (12k)
	H	8f	0.26	0.32 (12k)
H_3S - $R3m$	S	3a	0.02	0
	H	9b	0.11	0
$LaBH_8$ - $Fm\bar{3}m$	La	4b	0	0
	B	4a	0	0
	H	32f	0.20	0.25
H - $Cmca$-4	H	8f	0.29	0.22

Supplementary Table 8: **iCOBI based symmetry index.** The symmetry inequivalent atoms and their Wyckoff positions in the SB and AB structures along with the corresponding symmetry index computed treating the nuclei as classical, S_a^{c}, and quantum, S_a^{q}, objects calculated with a cutoff on the iCOBI of 0.05 Å. The Wyckoff parameters given in parenthesis for ScH_6 - $Cmcm$ correspond to the positions of these atoms in the higher symmetry ScH_6 - $P6_3/mmc$ phase.

Structure	Atom	Wyckoff letter	S_a^{c}	S_a^{q}
Symmetric Bonding				
PdH - $Fm\bar{3}m$ (1 atm)	Pd	4a	0	0
	H	4b	0	0
AlH_3 - $Pm\bar{3}m$ (135 GPa)	Al	2a	0	0
	H	6c	0	0
LaH_{10} - $Fm\bar{3}m$ (250 GPa)	La	4b	0	0
	H	8c	0	0
	H	32f	0.009	0.009
H - $I4_1/amd$ (500 GPa)	H	4b	0	0
PtH - $P6_3/mmc$ (100 GPa)	Pt	2d	0	0
	H	2a	0	0
YH_6 - $Im\bar{3}m$ (150 GPa)	Y	2a	0	0
	H	12d	0	0
H_3S - $Im\bar{3}m$ (250 GPa)	S	2a	0	0
	H	6b	0	0
Asymmetric Bonding				
ScH_6 - $P6_3/mmc$ (140 GPa)	Sc	2d	0	0
	H	12k	0.056	0.054
ScH_6 - $Cmcm$ (100 GPa)	Sc	4c	0.012	0 (2d)
	H	16h	0.040	0.055 (12k)
	H	8f	0.082	0.055 (12k)
H_3S - $R3m$ (130 GPa)	S	3a	0.076	0
	H	9b	0.116	0
$LaBH_8$ - $Fm\bar{3}m$ (100 GPa)	La	4b	0	0
	B	4a	0	0
	H	32f	0.062	0.063
H - $Cmca$-4 (450 GPa)	H	8f	0.022	0.014

Supplementary Table 9: **iCOBI based symmetry index with lower cutoff.** The symmetry inequivalent atoms and their Wyckoff positions in the SB and AB structures along with the corresponding symmetry index computed with the iCOBI, treating the nuclei as classical, S_a^{c}, and quantum, S_a^{q}, objects with a cutoff value of 0.018 Å. The lower cutoff produces the same qualitative result of the 0.05 Å cutoff.

Structure	Atom	Wyckoff letter	S_a^{c}	S_a^{q}
Symmetric Bonding				
PdH - $Fm\bar{3}m$	Pd	4a	0	0
	H	4b	0	0
AlH_3 - $Pm\bar{3}n$	Al	2a	0	0
	H	6c	0	0
LaH_{10} - $Fm\bar{3}m$	La	4b	0	0
	H	8c	0	0
	H	32f	0.011	0.009
H - $I4_1/amd$	H	4b	0	0
PtH - $P6_3/mmc$	Pt	2d	0	0
	H	2a	0	0
YH_6 - $Im\bar{3}m$	Y	2a	0	0
	H	12d	0	0
H_3S - $Im\bar{3}m$	S	2a	0	0
	H	6b	0	0
Asymmetric Bonding				
ScH_6 - $P6_3/mmc$	Sc	2d	0	0
	H	12k	0.034	0.033
ScH_6 - $Cmcm$	Sc	4c	0.012	0 (2d)
	H	16h	0.046	0.033 (12k)
	H	8f	0.023	0.033 (12k)
H_3S - $R3m$	S	3a	0.117	0
	H	9b	0.075	0
$LaBH_8$ - $Fm\bar{3}m$	La	4b	0	0
	B	4a	0	0
	H	32f	0.032	0.032
H - $Cmca$-4	H	8f	0.022	0.014

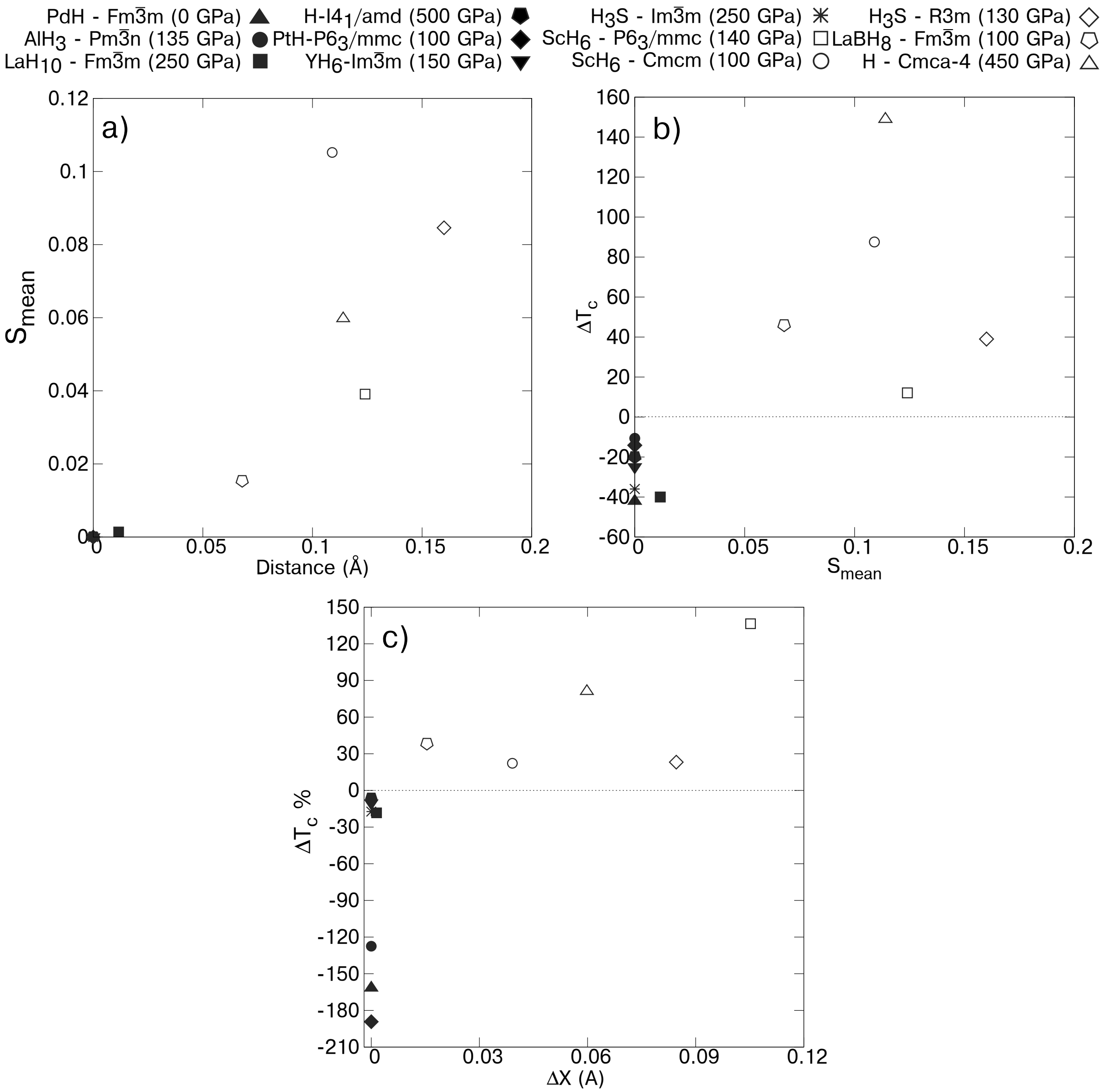

Supplementary Figure 3: **Symmetry index, Eliashberg function, and T_c variations.** (a) Classical mean value of S$_a$ ($S_{\rm mean} = (\sum_a N_a S_a)/(\sum_a N_a)$), calculated with the bond valence function. N_a represents the number of atoms in the ath Wyckoff site, as a function of the atomic displacement distance ($\Delta X = \sum_a^{N_A} \frac{|\mathcal{R}_q^a - \mathbf{R}_c^a|}{N_A}$), and (b) the variation of the T_c as a function of the classical mean value of S$_a$. (c) Percentage change of T_c as a function of ΔX. It can be observed that greater ΔX are reflected by greater changes in T_c. The panels highlight that the bond valence function method is able to identify a threshold beyond which the bonding environment is asymmetric enough for the non-local effects of quantum fluctuations to introduce structural distortions. Crucially, such distortions are not detrimental but instead lead to a positive shift in the T_c.

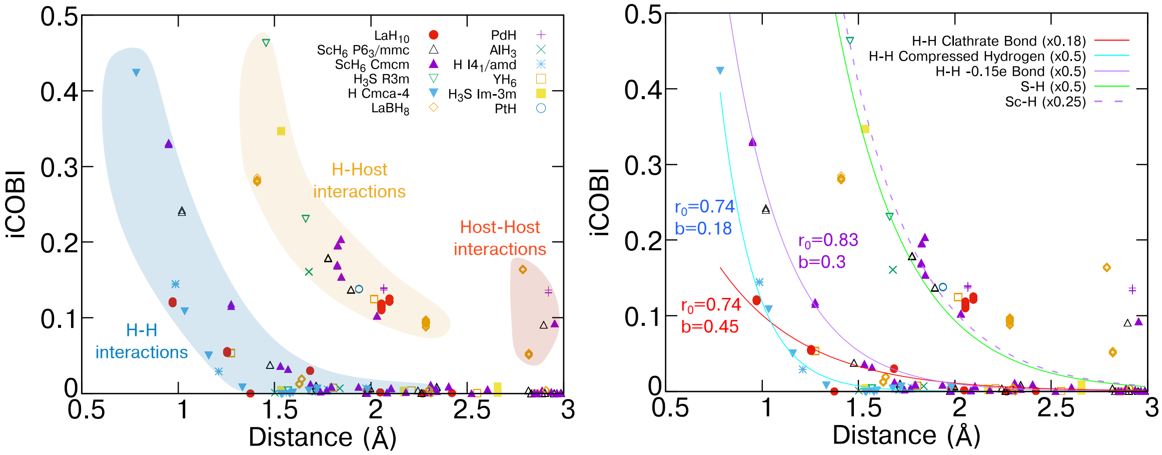

Supplementary Figure 4: **iCOBI interactions and bond valence function fits.** (Left) iCOBI values for all systems and pressures analyzed in this study. The colored areas highlight the types of interactions associated with the iCOBI values: blue for hydrogen–hydrogen, orange for hydrogen–host, and red for host–host interactions. (Right) Decay behavior and optimized parameters associated with the bond valence function (lines) overlapped with the iCOBI interactions (points). The red (clathrate hydrogen), cyan (compressed hydrogen), and continuous purple lines ($-0.15e$ doped hydrogen) correspond to different optimizations related to specific decays observed for the hydrogen–hydrogen interactions, while the green and dotted purple lines represent results obtained using literature values for the parameters employed for calculating the bond valence functions for the H–S and H–Sc interactions. The bond valence functions are scaled only for graphical overlap purposes.

References

(1) Deringer, V. L.; Tchougréeff, A. L.; Dronskowski, R. Crystal Orbital Hamilton Population (COHP) Analysis as Projected From Plane-Wave Basis Sets. *J. Phys. Chem. A* **2011**, *115*, 5461–5466.

(2) Müller, P. C.; Ertural, C.; Hempelmann, J.; Dronskowski, R. Crystal Orbital Bond Index: Covalent Bond Orders in Solids. *J. Phys. Chem. C* **2021**, *125*, 7959–7970.

(3) Coulson, C. A. The Electronic Structure of Some Polyenes and Aromatic Molecules. VII. Bonds of Fractional Order by The mMlecular Orbital Method. *Proc. R. Soc. Lond.* **1939**, *169*, 413–428.
